# Adsorptive performance of MWCNTs for simultaneous cationic and anionic dyes removal; kinetics, thermodynamics, and isotherm study

**DOI:** 10.3906/kim-2005-12

**Published:** 2021-08-27

**Authors:** Zaheer ASLAM, Irfan YOUSAF, Abdul ZAHIR, Adnan AKHTAR

**Affiliations:** 1 Department of Chemical Engineering, University of Engineering and Technology, Lahore Pakistan; 2 National Textile Research Centre, National Textile University, Faisalabad Pakistan; 3 Department of Chemical Engineering, Sharif College of Engineering and Technology, Lahore Pakistan

**Keywords:** Multi-walled carbon nanotubes, Congo Red, Rhodamine-B, adsorption, wastewater treatment

## Abstract

Disposal of contaminated wastewater causes many serious problems especially when it gets mixed with the ground and seawater. It is, therefore, important to apply any remedial action to eradicate dangerous pollutants from the aqueous effluents and to avoid exposure of this wastewater to aquatic life. The research results discussed herein deal with the removal of Rhodamine B (RhB) and Congo Red (CR) dye from wastewater by using multi-walled carbon nanotubes (MWCNTs) as an adsorbent. Different factors like solid dosage, initial pH and concentration, time, and temperature were studied to understand the behavior and mechanism of adsorption. The maximum adsorption capacity in case of a single component system was found to be 302 mg/g and 300 mg/g for Congo Red and Rhodamine B, respectively. Moreover, the mechanism of adsorption was best described by a pseudo-second-order kinetic model. Thermodynamic parameters showed that adsorption of CR and RhB was exothermic when these were removed from a single dye system. However, the overall process became endothermic for concurrent removal of both dyes from the solution. The research results showed that the MWCNTs could successfully be utilized to remove the dye from the industrial wastewater.

## 1. Introduction

Numerous types of dyes and pigments have been discharged into the aquatic environment through wastewater from various industries such as paper and pulp, textile, leather, and printing industries [1]. Wastewater from the textile dyeing operation contains unused dyes (~8% to 20% of the total pollution load) and auxiliary chemicals and significantly contribute to water pollution [2]. The presence of dyes in water imparts daunting effects in photosynthesis process as dye pollutants resist the supply of sunlight penetration [3]. Dyes are classified into a number of types owing to their structural variation and complex nature [4]. Cationic dyes, that can also be called as basic dyes, are used as a basic raw material in wide number of industrial applications including silk, nylon, acrylic, and dyeing of wool [5]. Due to the structure complexity, these dyes are resistant to degradation by conventional wastewater treatment techniques involving physicochemical and biological techniques [6]. Furthermore, degradation by a physical, chemical or biological mechanism would result in the generation of wide variety of intermediate species that can prove to be more toxic than parent dye compound [5,7].

Conventional methods to eradicate coloured compounds and organic contaminants from polluted streams include coagulation, flocculation, reverse osmosis, photo irradiation, ozonation process, and carbon adsorption [8–10]. Among these processes, the adsorption technique has widely practised due to its effectiveness, simplicity, high efficiency, and availability of a wide range of materials [11–15]. Carbon nanotubes (CNTs) are the latest type of carbon materials and it finds many applications in different areas of wastewater treatment and other engineering methods such as adsorption, separations, catalysis, in sensors, as biomedicine and synthesizing electronic devices [16–23]. Due to its larger specific surface area, small pore size, and hollow structural characteristics, carbon nanotubes have been proven to possess a great potential as a superior adsorbent for removing many kinds of organic and inorganic toxic components from the aqueous phase [24]. In most of the investigation done in past, dye adsorption studies onto carbon nanotubes were exclusively carried out for a single pollutant system and relatively less work is published narrating adsorption of dyes from a binary dye system. For real wastewater, multiple dyes are generally present and that versatilities may affect dye adsorption behaviour. Competitive effects usually modify the conduct of adsorption of each dye. Therefore, this research aims to study the dye adsorption from a multiple dye system onto high surface area nanomaterial. In view of the going further, the objective of this work is to investigate adsorption kinetics, isotherm, and thermodynamics for the treatment of polluted water. Different combinations of cationic and anionic test dyes were considered and experimental results were analyzed. 

## 2. Materials and methods

### 2.1. Reagents

An industrial-grade nonfunctionalized MWCNTs (Purity > 90 wt%, length: 10–30 m, Outer diameter: 10–30 nm) was purchased from Cheap Tubes Inc. USA and it was used without any further modification. Analytical grade Congo Red (C_32_H_22_N_6_Na_2_O_6_S_2_, molecular weight = 696.67 g/mol, λ_max_ = 498 nm, PK_a_= 3.5) and Rhodamine-B (C_28_H_31_ClN_2_O_3_, molecular weight = 472.02 g/mol, λ_max_ = 518 nm, PK_a_= 4.3) were purchased from Sinopharm Chemical Reagent Co. Ltd (Shanghai, China). The NaOH and HCl were bought from Merck (Darmstadt, Germany) and were used for adjusting the pH of dyes solution. One thousand ppm stock solutions of each pollutant dye were prepared by dissolving accurately weighing the required amount of CR and RhB. The single and binary dyes solution samples were obtained by appropriately mixing and diluting the stock solution to the desired concentration for subsequent experimentation. 

### 2.2. Material characterization 

Functional groups on the surface of material were determined by analysing it through fourier transform infrared spectroscopy (FTIR-4100-Jacso) using ZnSe ATR module within the IR range of 4000–650 cm^–1^. The surface area of MWCNTs was estimated through BET surface area analyzer (Micrometrics, ASAP 2020). The topology of the MWCNTs was observed using the field emission scanning electron microscope (Mira3, Tescan, UK) equipped with energy dispersive X-ray (x-act INCA, Oxford-USA) at accelerating voltage of 10 KV. To avoid the accumulation of electric charge on the sample, the specimen was coated with gold by placing the specimen in a sputter coater (AGB7340, Agar-UK) under vacuum for 1 min. Zeta potential of the MWCNTs was measured as a function of pH by differential light scattering technique (NanoZS, Malvern ZetaSizer-UK) using ethanol as an electrolyte. Homogeneous dispersions of MWCNTs were prepared by placing the dispersions in an ultrasonic liquid processor (VCX-750, Sonics-USA) for 5 h prior to the analysis. The buffer solutions of 0.1M HCl and 0.1 M NaOH were used to prepare dispersions of MWCNTs with different pH values (2–11).

### 2.3. Batch adsorption

All batch adsorption experiments with single as well as binary dye solution were carried out in an Erlenmeyer flask by taking 100 mL specimen solution. The test flasks were agitated at 200 rpm by fixing them on an orbital shaker. The effect of various parameters like MWCNTs dosage (0.025 g to 0.2 g), contact time of pollutant solution with adsorbent (1 min to 40 min), initial solution pH (2 to 9), initial concentration of dye solution and temperature (293 K to 323 K) were observed. For the co-adsorption of dyes from binary dye solution, the experiments were carried out by fixing the concentration of one dye and vary the concentration of other dye (100–400 ppm) and vice versa. After the equilibrium was achieved in a particular run, the solution was filtered out to separate the adsorbent from solution and filtrate was analyzed using a UV–vis spectrophotometer (JASCO Model V-6700). For the single dye systems, the remaining concentration of dye was evaluated by comparing the absorbance of solution (corresponding to λ_max_ of each dye) with calibration curve recorded earlier for pre-determined dye concentrations. For binary dye systems, absorption value was measured against both wavelength and unknown concentrations in solutions were determined by the following equations [25].

(1)C1=k22A1-k21A2k11k22-k12k21

(2)C2=k11A2-k12A1k11k22-k12k21

where k_11_, k_21_, k_12_ and k_22_ are the cross-calibration constants for components CR (1) and RhB (2) at the wavelength λ_1,max_, λ_2,max_, and their values are 0.039, 0.039, 0.026, and 0.217 respectively. A_1_ and A_2_ are the absorbance at wavelength λ_1,max_ and λ_2,max_, respectively. The adsorption capacity of each dye per unit weight of adsorbent at equilibrium, q_e_ (mg dye/g adsorbent) can be calculated by using the equation:

(3)qe=(C0-Ceq)V/W

where q_e_ is the adsorption capacity of a single or binary system and C_o_ and C_eq_ are the initial and the final equilibrium concentration of dye. V is the volume of the dye-containing solution (L) and w is the mass of the adsorbent (g). Moreover, thermodynamic selectivity of MWCNTs in the single and binary solution can be calculated by the following equation: 

(4)β=Qi,bxQj,sQj,bxQi,s

where Q_i,s_ and Q_i,b_ represents the amount of Congo Red adsorbed, i, in single and binary solution respectively. However, Q_j,s_ and Q_j,b_ are the amount of Rhodamine B adsorbed, j, in single and binary solutions respectively. 

## 3. Results and discussion

### 3.1. Characterization analysis

FTIR spectra of MWCNTs is shown in Figure S1. Presence of transmittance peak at 3746 cm^–1^ is due to the aromatic C-H stretching. However, the transmittance peaks at 1534 cm^–1 ^and 975 cm^–1^ in the fingerprint region exhibit the Sp^2^ hybridized -C=C and -C-C vibrational stretching in the MWCNTs structure, respectively. Absence of any other characteristic peak in the spectrum confirms that there is no organic impurity in the MWCNTs. The SEM micrographs in Figure S2 depicts that the MWCNTs are randomly oriented, highly tangled, and have a sharp nonporous tubular structure. The elemental analysis by energy dispersive X-ray (EDX) indicates the presence of only carbon as can be seen in Figure S3 which exhibits that there exists no surface impurity in MWCNTs. Figure S4 shows the BET analysis of the MWCNTs. The adsorption-desorption data of N_2_ gas was used to determine the surface area. The BET surface area of MWCNTs was 121.8 m^2^/g which is good for the adsorption process. Zeta potential results in Figure S5 show that pH ~6.7 corresponds to the isoelectric point (IEP) of MWCNTs. At pH < pH_IEP_ the surface will accumulate positive charge in contrast to pH > pH_IEP_ where the surface has net negative charge.

### 3.2. Influence of adsorbent dose

**Figure 1:**
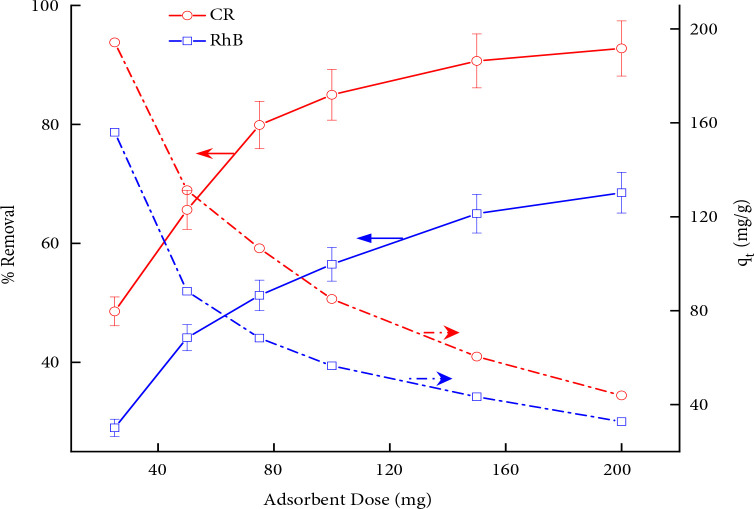
Adsorbent quantity versus %removal and uptake capacity at standard conditions (T = 303 K, pH = 6.5, C_o_ = 100 ppm, t = 100 min).

The adsorbent amount required to eradicate the pollutant from waste solution estimates the capacity of adsorbent and in turn cost-effectiveness of the process. The experimental results of the effect of dosage on the removal of dyes from aqueous solution bearing single pollutant dye are presented in Figure 1. It is indicated that higher dosage favours the removal of dyes because the greater amount of adsorbent provides more surface for attachment of dye molecules either by physical and/or chemical interaction with the surface. The acidic CR has a good affinity toward the MWCNTs when compared to the basic dye RhB. At a particular dosage, the CR removal is higher compared to RhB probably because of more negatively charged functional groups (-SO_2_Na) in the CR dye as compared to RhB (∅-N^+^_-_X). Based on the effect of adsorbent dose data 100 mg dosage was selected for subsequent experiments for determining the percent removal and the adsorption capacity. 

### 3.3. Kinetic study

**Figure 2:**
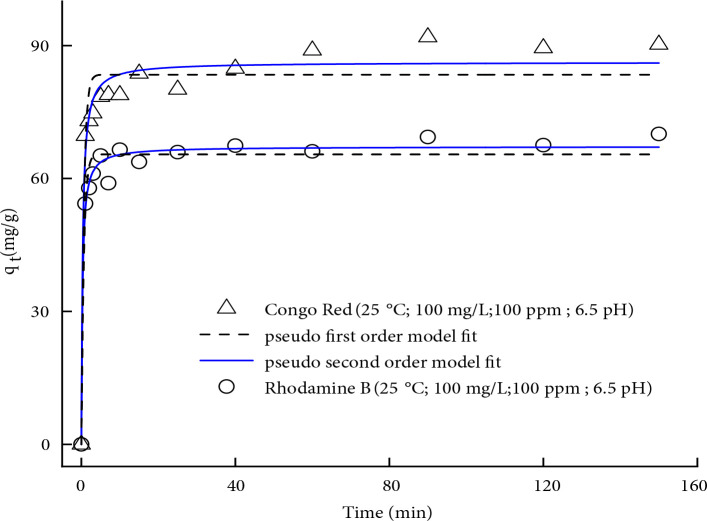
Adsorption kinetics of Congo red (CR) and Rhodamine B (RhB) on MWCNTs (T = 303 K, initial solution pH = 6.5, C_o_ = 100 ppm, t = 100 min)

Optimal adsorption time is a significant parameter to ensure complete equilibrium between pollutants and adsorbent. The influence of contact time on the uptake capacity of MWCNTs is shown in Figure 2. It is clearly noticed from the measured concentration versus time profile that both CR and RhB dye pollutants attain equilibrium at ~86 mg/g and ~65 mg/g respectively in less than 10 min and no significant change was observed after it. Equilibrium adsorption time is a key factor to study the adsorption kinetics [26,27]. Adsorption kinetics provides useful information regarding the mechanism of adsorption which is necessary for optimization and feasibility study of a sewage treatment process. The adsorption kinetic data were modelled by pseudo-first-order (Lagergren-first-order) and pseudo-second-order kinetic equations. The best fit for each model was evaluated based on the value of the regression coefficient (R^2^). The nonlinear pseudo-first-order kinetic equation can be expressed as follows:

(5)qt=qe(1-e-ek1t)

where q_t_, (mg/g) is the adsorbed amount of pollutant per unit mass of adsorbent at any instant of time “t” and at equilibrium respectively, (min^–1^) is the rate constant of the pseudo-first-order kinetic model. Pseudo-second order model can be expressed by the following equation:

(6)qt=qe2k2t1+qek2t

where is k_2_ the pseudo-second-order rate constant (g/mg min). Nonlinear curve fitting of both models was performed using Origin Lab (V.9.1.0) software. Parametric values of kinetic models were obtained by nonlinear curve fitting and are summarized in Table 1. It can be noticed that the for the pseudo-first and second-order model is 0.94 and 0.97 for CR respectively, and its value for RhB is 0.96 and 0.98, respectively. Therefore, it can be concluded that the pseudo-second-order model best describes the adsorption dynamics of both dyes onto MWCNTs. This also supports that the adsorption of CR and RhB onto MWCNTs is either due to attractive forces exerted by the valence shell electrons or as a result of sharing and exchange of electrons between both parties, i.e. adsorbent and adsorbate [28]. Moreover, the equilibrium adsorption capacity calculated from pseudo-second-order kinetic model is much closer to the experimental uptake capacity as can be seen in Table 1. 

**Table 1:** Kinetic parameters of CR and RhB dyes adsorption.

Model	Parameters	Congo Red	Rhodamine B
First order model	K1 (L/mg)	1.58	1.57
	Qe (mg/g)	83.81	65.44
	R2	0.94	0.96
Second order model	K2 (L/mg)	0.034	0.054
	Qe (mg/g)	86.71	67.19
	R2	0.97	0.98

### 3.4. Influence of initial concentration of dyes

The effect of initial CR and RhB concentration in a binary mixture on the uptake capacity of MWCNTs was investigated and compared it with adsorption of dyes from single dye solution. The initial concentration of one dye was varied from 100 to 400 mg/L by fixing the concentration of other dye at a constant temperature, volume, and pH. The resultant uptake capacities are summarized in Table 2. The interactions of multiple pollutants may mutually enhance or inhibit the adsorption capacity during the treatment of wastewater by adsorption process. It can be noticed from Table 2 that the individual uptake as well as the selectivity at equilibrium, is a function of the initial concentration of dye in the polluted solution. Adsorption results of CR from a single dye solution shows the increase in uptake capacities from 85 mg/g to 352.7 mg/g when initial CR concentration varies from 100 to 400 mg/L. However, in the same range of initial concentration, the adsorption capacity of RhB (changed from 68.1 mg/g to 316.5 mg/g) is little low than CR. Comparing the adsorption of single and binary component solutions, it can be seen that the individual capacities of each dye in a binary component system were lower than those in a single component system. For instance, without RhB, the uptake for 300 mg/L of CR was found as 259 mg/L. But the inclusion of RhB in the solution having 100, 200, 300, 400 mg/L, decreases the sorption capacity of CR 174.4, 162, 138.1, and 114.6 mg/g respectively. The adsorption from binary solution mostly shows the antagonistic effect hence lower uptake of the solid adsorbent. However, at lower concentrations in the binary dye system, synergistic sorption (q_i,mix_/q_i_ > 1) can be observed when concentrations of both dyes were fixed at 100 mg/L as shown in Table 2. During the adsorption from a binary solution, a competition evolves for adsorption binding sites present on the surface and some of the sites are filled by the other competing dye, especially at high concentration. The values of the selectivity factor for CR/RhB system tends to decrease with rising corresponding initial concentrations of pollutant dyes. The “β” ranges from 1.09 to 0.54. It can be noticed from the table that the difference in consecutive values of β at low concentrations of CR is large however it diminishes at the relatively higher initial load of CR dye in the binary system. This may be associated to possible π-π stacking of competing dye molecules in addition to interaction with adsorbent surface and resultant increase in overall uptake capacity of the adsorbent. 

**Table 2:** Comparison of the individual equilibrium adsorption uptake of CR and RhB in single and binary dye solutions onto MWCNTs (T = 303 K, pH = 6.5, dosage = 100 mg, t = 100 min).

(mg/L)	(mg/L)	(mg/g)	(mg/g)			Selectivity factor, β
0	100	0	76.1			
0	200	0	160.9			
0	300	0	237.8			
0	400	0	270.5			
100	0	85	0			
200	0	176.7	0			
300	0	259	0			
400	0	297.6	0			
100	100	92.3	84.9	1.1	1.1	0.5
100	200	61.1	167.6	0.7	1.0	2.4
100	300	52.6	208.9	0.6	0.9	1.6
100	400	37.6	254	0.4	0.9	1.7
200	100	124.6	76.5	0.7	1.0	1.6
200	200	149.6	141.2	0.9	0.9	0.9
200	300	75.6	210.5	0.4	0.9	2.0
200	400	106.8	222.4	0.6	0.8	1.0
300	100	174.4	74.1	0.7	1.0	1.6
300	200	162	139	0.6	0.9	1.5
300	300	138.1	191.9	0.5	0.8	1.5
300	400	114.6	207.3	0.4	0.8	1.3
400	100	188.4	78	0.6	1.0	2.4
400	200	161	126.1	0.5	0.8	1.6
400	300	161.3	191.7	0.5	0.8	1.7
400	400	176.5	191.7	0.6	0.7	1.1

### 3.5. Equilibrium isotherm modelling

The adsorption equilibrium isotherm is important to study how the adsorbate is distributed between liquid and solid phases and reaches equilibrium. The empirical equation derived from isotherm data is fundamental in describing the interactive behaviour of pollutant and adsorbent to optimize the design economics for scale-up of the separation process. The parameters like maximum adsorption capacity, nature of adsorption (homogeneous or heterogeneous), and separation constant help to determine interaction as well as the mechanism of adsorption process. Various theories and empirical models have been reported in this regard such as Langmuir, Freundlich, and Sips.

The Langmuir adsorption isotherm model is valid for the assumption that the surface coverage of solid is homogeneous (i.e. adsorbent has a finite capacity for the adsorbate and all binding sites are identical and energetically equivalent). Therefore, the Langmuir isotherm model is best to calculate the monolayer capacity of solid surface. The nonlinear form of the Langmuir isotherm model can be written as follows;

(7)qe=qmaxKLCe1+KLCe

where C_e_ (mg/L) and q_e_ (mg/g) are equilibrium liquid-phase concentration of dye and adsorbed amount respectively, q_max_ is the monolayer capacity, K_L_ (L/mg) the Langmuir constant and is related to apparent energy of adsorption. The Freundlich isotherm model is applicable for multilayer adsorption and is derived by assuming a heterogeneous surface with nonuniform distribution of surface energy [29]. The formal equation of Freundlich isotherm is given below;

(8)qe=KFCe1nf

where K_F_ and n_f_ are isotherm constants indicating the capacity and intensity of the adsorption. The higher value of K_F_ indicates a higher affinity of the adsorbent for the adsorption of dye molecules and the factor 1/n_f_ indicates the heterogeneity of surface and as the value gets closer to 0 the surface of the adsorbent exhibits more heterogeneous adsorption behavior and it also determines the favorability of the adsorption process [30]. Sips isotherm incorporates the features of both Langmuir and Freundlich isotherm models [31]. The specific equation is given by

(9)qe=qmaxKsCens/1+KsCe

where K_S_ (L/mg) is the Sips model isotherm constant and n_s_ is heterogeneity constant. 

The agreement among experimental and theoretical data points after isotherm models curve fitting is shown in Figure S6. The statistical indices obtained from a nonlinear regression (performed by using Origin Pro version 9.1.0) of each isotherm model and their pertinent variables for both CR and RhB dyes adsorption from a single component solution are shown in Table 3. As a consequence, it can be predicted that the adsorption of both pollutants is inhomogeneous over the adsorbent surface. Based on lowest RSS and R^2^ values the isotherm equations fitted for CR and RhB can be indicated in the order (Sips’ > Langmuir > Freundlich). Langmuir model constant reflects the affinity for binding of component dye onto MWCNTs [32]. The K_a_ value for CR is greater than that of RhB which implies strong bonding of CR with CNTs surface. The value of “n” greater than unity in Freundlich isotherm indicates that the adsorption is favourable and higher magnitude of K_F_ exhibits relatively easy uptake of CR as compared to RhB. The correlation coefficients of the Sips isotherm model reinstate that the adsorbent surface is a blend of homogenous and inhomogeneous sites with 301.3 mg/g and 300 mg/g maximum adsorption capacity of CR and RhB respectively as seen in Table 3. Prediction of binary-component isotherm data has always been complex due to its interactive and competitive effects as compared to single component equilibrium data. The simultaneous removal of RhB and CR onto MWCNTs from binary pollutant solution was investigated by the extended Langmuir isotherm model. This model assumes (i) each adsorption site possesses equal energy of adsorption (ii) each site will have a tendency to adsorb one dye at a time and not both dyes at the same time (iii) there exist no interaction of any sort between adsorbed molecules of adjacent sites. The generalized form of extended Langmuir equation is as follows [33,34]:

**Table 3:** Isotherm parameters for mono-component adsorption of CR and RhB onto MWCNTs.

Isotherm	Parameter	Pollutant dye	
		CR	RhB
Langmuir	qL,max (mg/g)	380	380
	KL (L/g)	0.035	0.02
	R2	0.925	0.924
	RSS*	3379.20	2865.83
Freundlich	KF (L/g)	40.02	21.04
	n	2.2	1.85
	R2	0.88	0.89
	RSS*	5342.41	3911.88
Sips	qS,max(mg/g)	302	300
	Ks (L/g)	3.01E-4	2.47E-4
	n	2.67	2.3
	R2	0.999	0.989
	RSS*	0.345	263.84

(10)qe,i=q0,iKiCe,i1+Σi=1nKiCe,i

where the q_e,i_ (mg/g) and C_e,i_ (mg/L) is solid and aqueous phase adsorbate concentration at equilibrium respectively for component “i”. The Langmuir model constants are q_0,i_ (mg/g) and K_i_ (L/g).

The extended Langmuir isotherm parameter and RMSE values for both CR and RhB are summarized in Table 4. The extended Langmuir model is adequately fitted to experimental values with the lowest error value 7.54 for CR and 5.031 for RhB. Inclusion of a second dye in the aqueous solution enhances the competitive and interactive effects existing in multicomponent systems and the extended Langmuir model constant gets negative value in the regression results. This is probably because of the interaction of dyes among each other which is a deviation from the assumption of the extended Langmuir model. Adsorption from a binary component solution may have three types such as synergistic sorption (i.e. q_i,mix_/q_i_ > 1), antagonistic behaviour (i.e. q_i,mix_/q_i_ < 1), and noninteractive sorption (i.e. q_i,mix_/q_i_ = 1). Where q_i,mix_ is the sorption capacity of the component in the presence of competing dye while the q_i_ is the uptake of dye when it exists alone in the solution. In this study, the ratio of q_i,mix_/q_i_ for RhB is higher than the corresponding CR. The ratio for RhB is closer to 1 at lower concentrations of CR however it reduces as the concentration of CR was increased in the binary solution. Overall, this ratio is lower in CR than for RhB as shown in Table 4. 

**Table 4:** Influence of increasing initial concentration of competing dye on the extended Langmuir isotherm constants of CR and RhB adsorption from binary dye systems.

		Extended Langmuir model parameters			Regression parameters	
Component	Co, RhBmg/L	q0,CRmg/g	K1min–1	K2min–1	R2	RMSE*
CR	100	184.7	1.559	0.911	0.935	27.10
	200	146.0	0.009	–0.017	0.993	8.55
	300	204.0	0.001	–0.008	0.991	8.69
	400	59.8	0.002	–0.006	0.994	7.54
Component	Co, RhBmg/L	q0,CRmg/g	K1min–1	K2min–1	R2	RMSE*
RhB	100	257.8	0.028	–0.012	0.993	11.855
	200	288.6	0.011	–0.005	0.996	8.437
	300	151.9	0.005	–0.007	0.998	5.031
	400	247.1	0.025	0.001	0.961	22.619

### 3.6. Thermodynamics of single/binary dye adsorption

The adsorption experiments with varying temperature were carried out to reveal the nature of the process of removal of CR and RhB dye onto MWCNTs as single and binary dye system. Thermodynamic parameters such as change in enthalpy (ΔH°), Gibbs (ΔG°) energy, and entropy (ΔS°) were determined by Vant Hoff’s equation given below 

(11)lnKa=-Δ GRT=-Δ HRT+Δ SR

where R is the gas constant (8.314 J/(mol K) ) and T is the temperature (K). K_a_ is the equilibrium constant obtained by the ratio of adsorbed pollutant concentration (C_s_) to the equilibrium concentration of dye pollutant in the solution (C_e_). The plot of Vant Hoff equation based on experimental data is presented in the Figure S7 and the thermodynamic parameters obtained from Figure S7 are summarized in Table 5. Negative values of Gibbs (ΔG) energy at all four temperatures shows that the adsorption is feasible and spontaneous for both pollutant dyes. As a general rule, ΔG for physisorption lies in the range –20 kJ/mol to 0 kJ/mol, however value in between –20 kJ/mol to –80 kJ/mol reflects physisorption along with chemisorption and values between –80 kJ/mol to –400 kJ/mol) can be viewed as adsorption process that involves chemisorption only [35]. In this research, calculated ΔG from experimental data lies in the physisorption range. The absolute values of ΔG are relatively higher in the binary system as compared to the single system which may be associated to interaction of counter charges on the dye moieties in addition to sorbate-sorbent interaction [36]. Increase in temperature raises the kinetic energy of dye and the probability of their collision with the sorbent surface and with their self and as a result pollutant removal increases. The negative values of ΔH for single system indicate the exothermic nature of adsorption of both dyes. But the process becomes reverse (ΔH is positive) for the binary dye system as can be seen in Table 5. Positive ΔH can be attributed to electrostatic interactions between the dyes which may, otherwise, require a higher energy barrier for the diffusion into the surface of adsorbent. For the dye ions to transport through the solution and reach active sites, it is imperative to strip out of their hydration shell that needs energy input. If interaction force linked to adsorption of dyes do not surpass the dehydration energy of dye ions, the overall energy balance will lead to endothermic behavior [37]. ∆S is the measure of randomness at the solid-solution interface due to distribution of adsorbate dyes onto adsorbent surface. ∆S is higher in case of dye adsorption from binary solution as compared to adsorption of dyes from single dye solution. This is probably because of greater randomness on MWCNTs surface due to synergism between counter dye ions. 

**Table 5:** Thermodynamic parameters for single and binary dye adsorption (dosage = 100 mg, t = 100 min, pH = 6.5, concentration for single dye solution = 100 ppm).

Pollutant system	Congo Red (CR)					Rhodamine-B (RhB)			
Single dFS1ye	T (K)								
	293	16.71	–6.86	-32.51	-16.36	5.6	–4.18	–36.42	–14.86
	303	12.80	–6.42			4.8	–3.94		
	313	11.31	–6.31			3.4	–3.21		
	323	8.70	–5.81			3.3	–3.22		
Binary (CR:RhB)									
60:100	293	5.4	–4.12	166.61	44.81	3.9	–3.30	48.49	10.82
	303	8.8	–5.47			4.9	–4.00		
	313	17.1	–7.38			5.3	–4.36		
	323	29.1	–9.05			5.9	–4.78		
100:60	293	11.4	–5.93	79.98	17.64	3.7	–3.15	87.83	22.51
	303	12.5	–6.36			5.1	–4.12		
	313	17.6	–7.46			7.3	–5.18		
	323	21.5	–8.24			8.4	–5.71		
100:100	293	6.3	–4.48	66.54	15.0	4.4	–3.59	40.89	8.37
	303	7.9	–5.21			5.0	–4.03		
	313	9.3	–5.81			5.6	–4.49		
	323	11.2	–6.49			6.0	–4.80		

### 3.7. Influence of PH on adsorption

**Figure 3:**
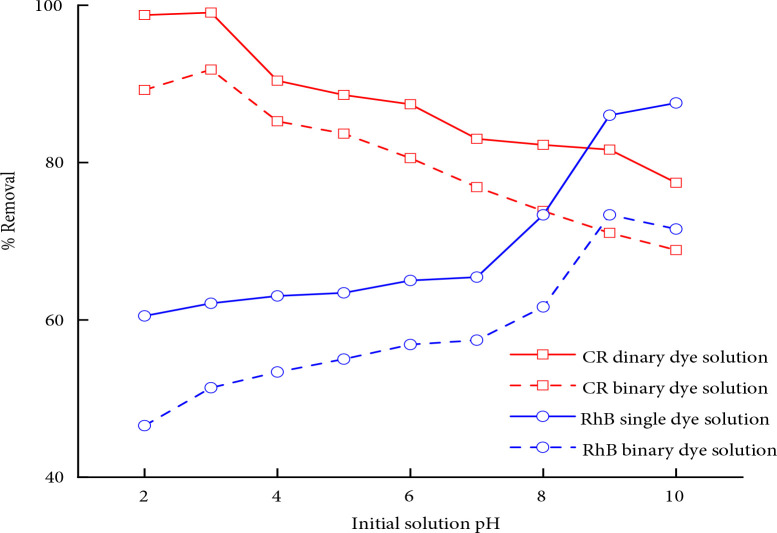
Adsorbent quantity versus %removal and uptake capacity at standard conditions (T = 303 K, pH = 6.5, C_o_ = 100 ppm, t = 100 min).

The effect of pH for the competitive adsorption of CR and RhB onto MWCNTs was studied at optimum dosage and time and results are presented in Figure 3. The adsorption of both dyes, i.e. CR and RhB can be explained on the basis of isoelectric point of the adsorbent. As the surface of MWCNTs tends to possess a positive charge at pH > pH_IEP_ (pH < 6.7). It can be noticed that the uptake capacity of CR increases with a decrease in initial solution pH and the maximum is observed corresponding to pH 3 in both single and binary dye solution. By lowering the solution pH, the uptake capacity of CR decline which is ascribed to the protonation of CR at pH < pKa (3.5 ± 0.5). Higher adsorption of CR can be associated to the two anionic functional groups (-SO_3_Na) in its chemical structure which are possibly attracted by the positively charged surface of MWCNTs at pH < 6.7. In comparison to CR, the competing dye (i.e. Rhodamine B) shows the reverse behavior as shown in Figure 3. Decrease in adsorption capacity from pH 4 to 2 is probably due to protonation of RhB at pH < 4.3. Moreover, being cationic in nature, RhB has low adsorption in acidic solution because of electrostatic repulsion between the positively charged surface of MWCNTs and dyes molecules [38,39]. At pH > pH_IEP_, rise in adsorption capacity of RhB is due to electrostatic attraction between the dye ions and the negatively charged adsorbent surface. The uptake capacity of different carbon based nanomaterials for the abatement of various dyes are compared with MWCNT’s used in this research and data is tabulated as Table 6. It can be concluded from the comparison table that MWCNTs could be practised for the treatment of dye laden wastewater. 

**Table 6:** Effect of initial solution pH (dosage = 100 mg; t = 100 min; T = 303 K, single dye solution concentration = 100 ppm, binary dye solution concentration = 100 CR:100 RhB).

Adsorbent	Pollutant	Uptake capacity (mg/g)	References
CNTs	Procion Red MX-5B	29.94	[40]
CNTs	C.I. Direct Yellow 86 (DY86)C.I. Direct Red 224 (DR224)	56.2 (DY86)61.3 (DR224)	[41]
MMWCNT’s	Methylene Blue (MB)Neutral Red (NR)Brilliant Cresyl Blue (BCB)	15.74 (MB)20.33 (NR)23.55 (BCB)	[42]
Fe3O4@graphene composite (FGC)	Methylene Blue (MB)Congo Red (CR)	45.27 (MB)33.66 (CR)	[43]
MWCNTs	Alizarin Red S (ARS)Morin	161.290 (ARS)26.247 (Morin)	[44]
MWCNTs	Congo Red	302	This study
	Rhodamine B	300	This study

## 4. Conclusion

In this research study, MWCNT showed considerable adsorption of CR and RhB. In single or binary component systems, CR adsorption was generally higher than RhB due to its anionic nature. Kinetic studies showed that CR and RhB adsorption followed a pseudo-second-order kinetics in single dye solutions. So, the pseudo-second-order model is the rate controlling step during the sorption process. On the basis of correlation coefficient R^2^, Sips model, and extended Langmuir model fits best for single and binary system, respectively. In the thermodynamic study, the negative values of indicated the spontaneous nature of adsorption and the positive values of and showed the endothermic nature and affinity of MWCNTs for CR and RhB, respectively. Effect of parameters including adsorbent dosage, pH, and initial concentration of dye was also investigated and results showed that the adsorption of RhB increases with an increase in pH while the CR showed an entirely different adsorption trend. Based on the acquired data, the studied adsorbent can be effectively used as an adsorbent for industrial wastewater treatment. 

Supplementary Materials
