# Supplementary material for: Adsorptive performance of MWCNTs for simultaneous cationic and anionic dyes removal; kinetics, thermodynamics, and isotherm study
Source: Turk J Chem. 2021 Aug 27;45(4):1189–200. doi: 10.3906/kim-2005-12 (PMC8517491; doi:10.3906/kim-2005-12)
Supplement: Supplementary file 1 — Supplementary Materials [file turkjchem-45-1189-sup001.pdf]

Supplementary material

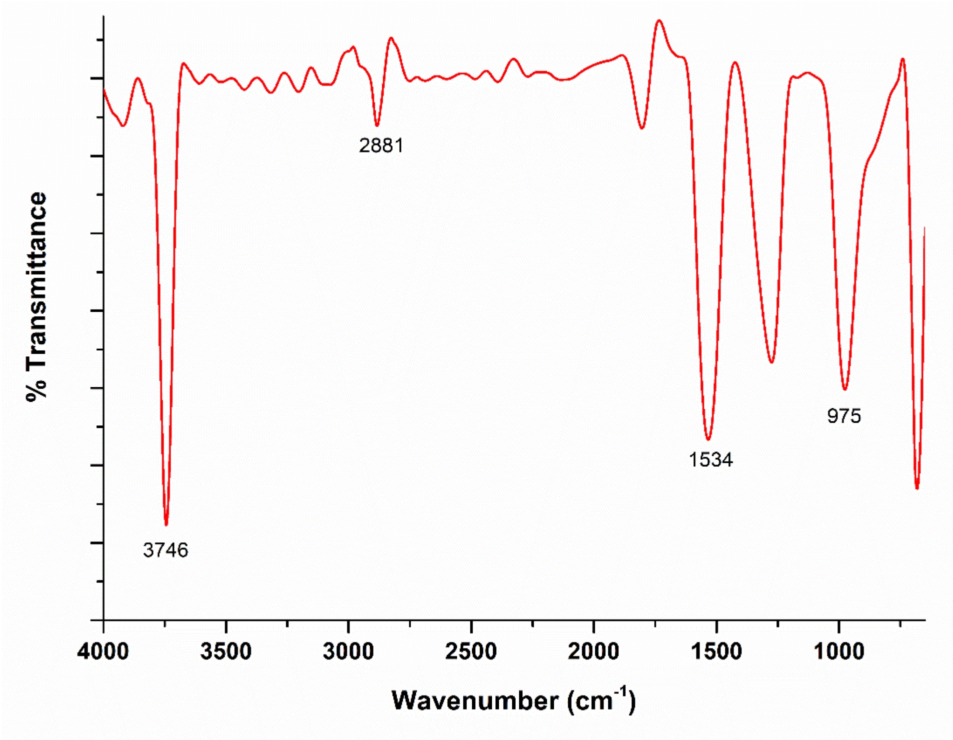

Figure S1. FTIR spectrum of MWCNTs.

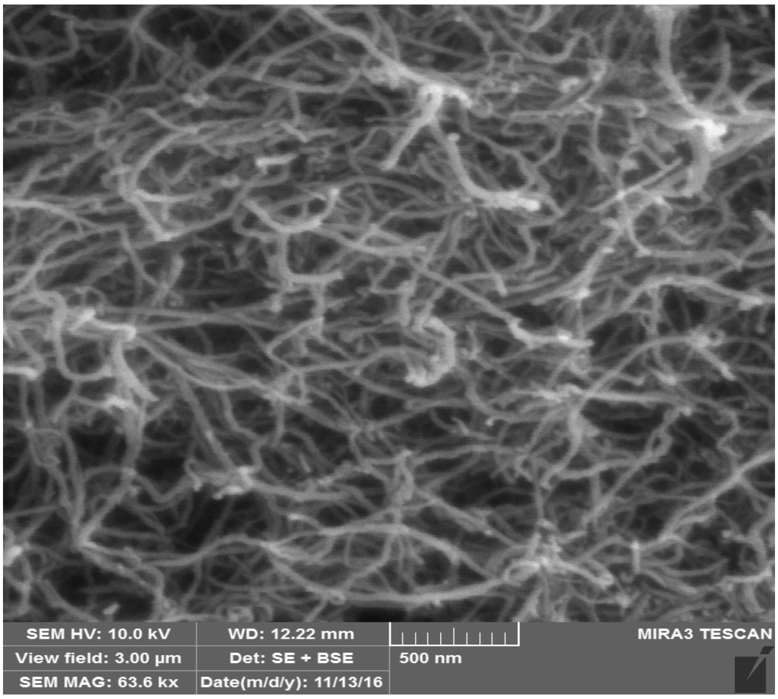

Figure S2. SEM image of MWCNTs.

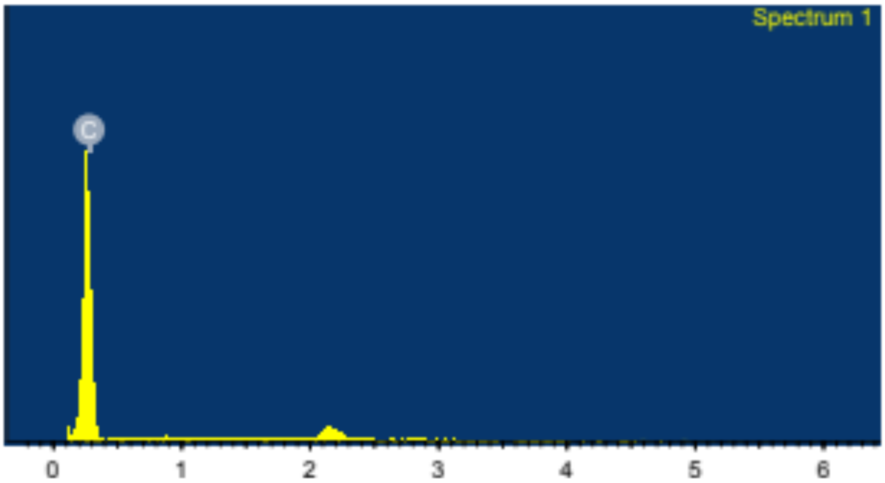

Figure S3. EDX spectrum of MWCNTs.

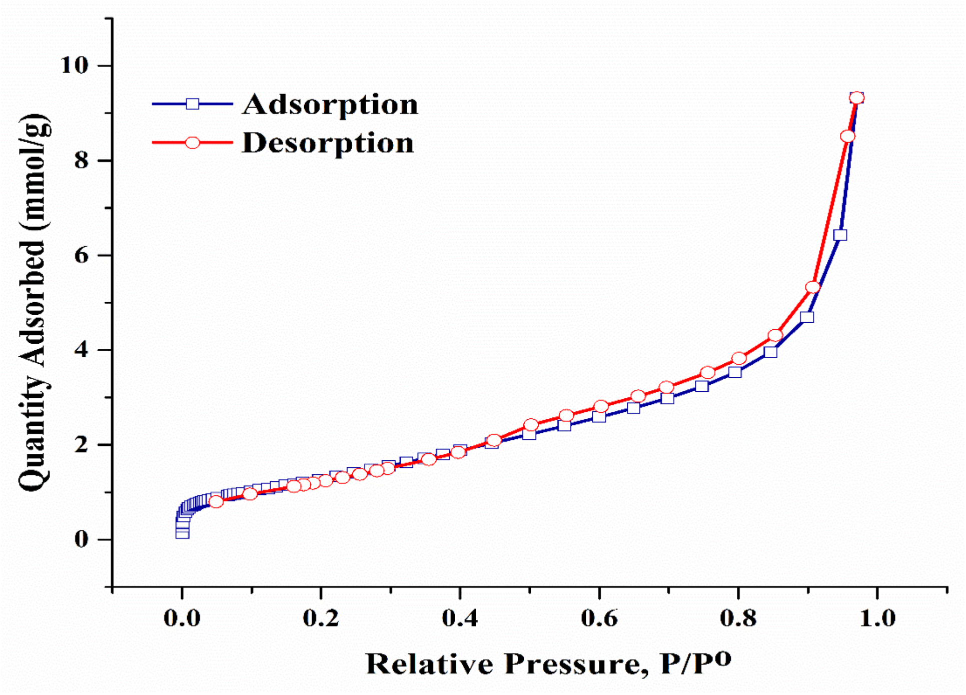

Figure S4. N_2 adsorption-desorption curves of MWCNTs.

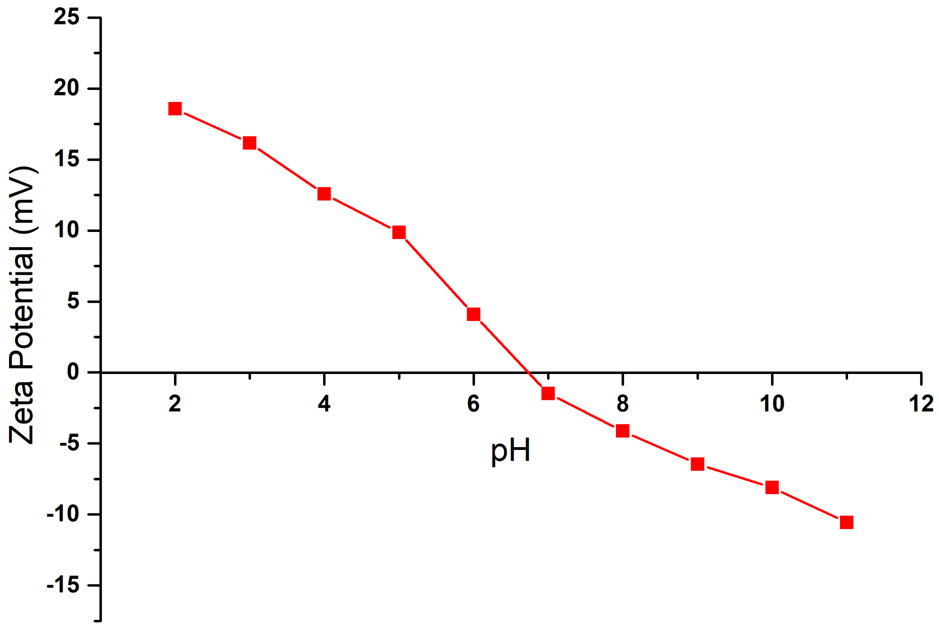

Figure S5. Zeta potential of MWCNTs.

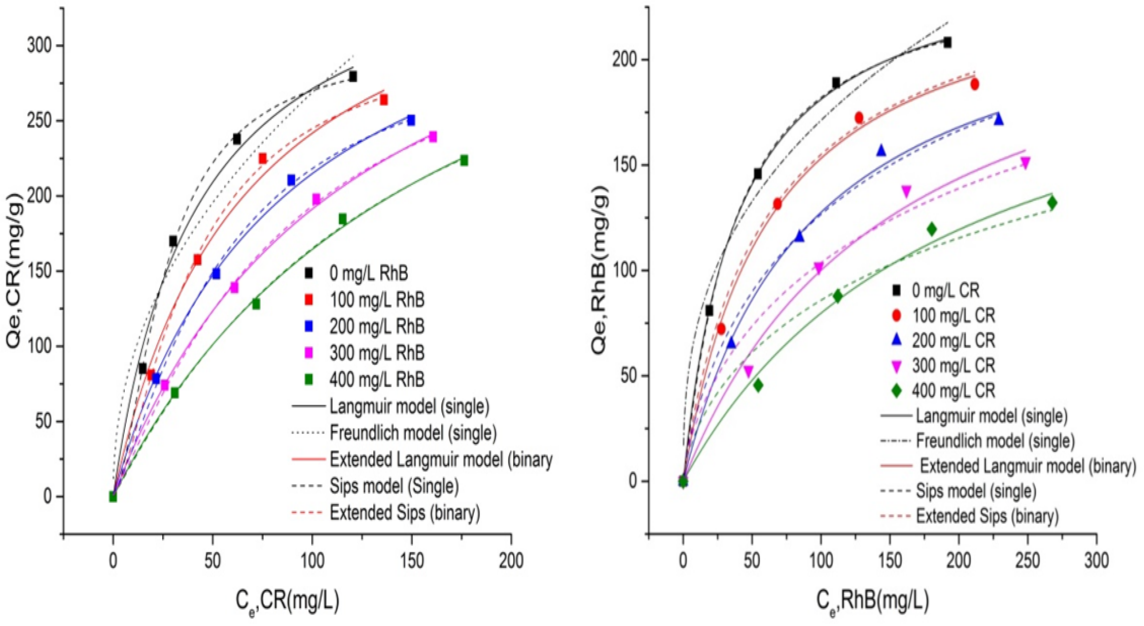

Figure S6. Adsorption isotherms for CR and RhB onto MWCNTs in single/binary dye system (dosage = 100 mg, time= 100 mint, V= 100 mL, T = 303 K, pH= 6.5).

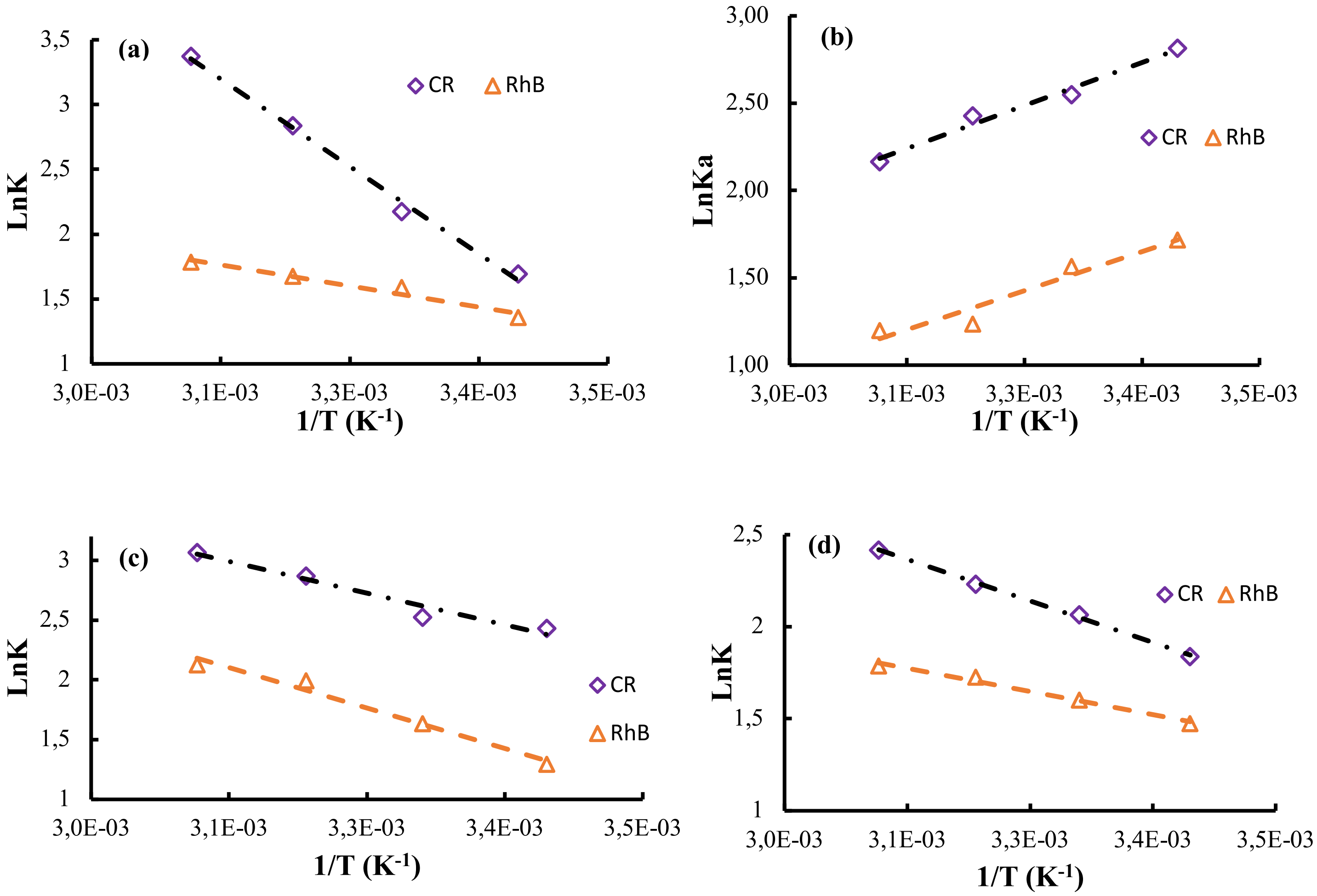

Figure S7. Von't Hoff plots (dosage = 100 mg, time = 100 min, pH= 6.5) for dye adsorption in single and binary dye solution (a) Single dye solution CR=100 ppm, RhB= 100 ppm, (b) CR:RhB = 60:100, (c) CR:RhB = 100:60 and (d) CR:RhB = 100:100.
